# The Quest for Anti-inflammatory and Anti-infective Biomaterials in Clinical Translation

**DOI:** 10.3389/fbioe.2016.00071

**Published:** 2016-09-09

**Authors:** May Griffith, Mohammad M. Islam, Joel Edin, Georgia Papapavlou, Oleksiy Buznyk, Hirak K. Patra

**Affiliations:** ^1^Department of Clinical and Experimental Medicine (IKE), Linköping University, Linköping, Sweden; ^2^Department of Neuroscience, Swedish Medical Nanoscience Center, Karolinska Institutet, Stockholm, Sweden; ^3^Department of Ophthalmology, Maisonneuve-Rosemont Hospital Research Center, University of Montreal, Montreal, QC, Canada; ^4^Department of Eye Burns, Ophthalmic Reconstructive Surgery, Keratoplasty and Keratoprosthesis, Filatov Institute of Eye diseases and Tissue Therapy of the NAMS of Ukraine, Odessa, Ukraine

**Keywords:** biomaterials, clinical translation, anti-inflammatory, anti-infective

## Abstract

Biomaterials are now being used or evaluated clinically as implants to supplement the severe shortage of available human donor organs. To date, however, such implants have mainly been developed as scaffolds to promote the regeneration of failing organs due to old age or congenital malformations. In the real world, however, infection or immunological issues often compromise patients. For example, bacterial and viral infections can result in uncontrolled immunopathological damage and lead to organ failure. Hence, there is a need for biomaterials and implants that not only promote regeneration but also address issues that are specific to compromised patients, such as infection and inflammation. Different strategies are needed to address the regeneration of organs that have been damaged by infection or inflammation for successful clinical translation. Therefore, the real quest is for multifunctional biomaterials with combined properties that can combat infections, modulate inflammation, and promote regeneration at the same time. These strategies will necessitate the inclusion of methodologies for management of the cellular and signaling components elicited within the local microenvironment. In the development of such biomaterials, strategies range from the inclusion of materials that have intrinsic anti-inflammatory properties, such as the synthetic lipid polymer, 2-methacryloyloxyethyl phosphorylcholine (MPC), to silver nanoparticles that have antibacterial properties, to inclusion of nano- and micro-particles in biomaterials composites that deliver active drugs. In this present review, we present examples of both kinds of materials in each group along with their pros and cons. Thus, as a promising next generation strategy to aid or replace tissue/organ transplantation, an integrated smart programmable platform is needed for regenerative medicine applications to create and/or restore normal function at the cell and tissue levels. Therefore, now it is of utmost importance to develop integrative biomaterials based on multifunctional biopolymers and nanosystem for their practical and successful clinical translation.

## Introduction

Transplantation with donor organs or tissues is often the only treatment available for patients with end-stage organ damage leading to failure. However, the demand for donor organs is manyfold higher than the supply of donated organs. According to US Department of Health and Human Service, an average of 79 people receive organ transplants each day while 22 others die while waiting to be transplanted, due to the acute shortage of high quality-donated organs.^1^ Even if donor organs are available, problems such as graft-versus-host disease and rejection leading to graft failure still need to be solved. An alternative to donor organ transplantation is use of bioengineered artificial organs or multicomponent tissues as replacements. Tissue engineering and regenerative medicine are, therefore, rapidly growing areas (Stock and Vacanti, 2001).

In many cases, organ failure is due to congenital defects or aging. However, infections caused by viruses and bacteria can cause uncontrolled damage leading to organ failure. Current examples include pandemics such as the severe acute respiratory syndrome (SARS) that broke out in 2003. SARS was caused by a coronavirus and patients who died were otherwise healthy young adults whose immune system mounted an inflammatory response resulting in the destruction of pulmonary stem cells that led to death of the patients (Holmes, 2003). Even in the common influenza, in severe cases, upregulation of matrix metalloproteinase (MMP)-9 has been shown to result in tissue destruction in various organs (Wang et al., 2010). Bacterial infections often progress in a slower manner than viral ones. One area where bacterial infections are particularly problematic is in chronic skin wounds, such as non-healing ulcers and skin burns. In the eye, ulcers and burns along with infections are also a source of inflammation and tissue destruction. Whether the infections and ensuing immunopathological mechanisms that are triggered cause acute and rapid organ destruction or chronic problems, these are considerations in the development of biomaterials as scaffolds or implants that are targeted to promoting regeneration under these compromised conditions. There is also a concern of biomaterials, which themselves have been associated with infection and inflammation.

Here, we review the biomaterials that are designed for use in compromised patients with inflammation or active infections. We present several examples of each of the groups of biomaterials, taken from our own research and cases that are documented within the literature.

## Biomaterials Associated with Undesired Inflammation and Infection

### Biomaterials and Inflammation

Biomaterials are essentially foreign to the human body and, as such, have been associated with triggering inflammation and immune reactions. Initial inflammation is necessary for wound healing and occurs when biomaterials are in contact with host tissues. The milder irritation produced includes mild to moderate pain or discomfort, such as itching. Inflammation occurs with a more severe response and presents redness, heat, swelling, pain. This is a defensive response and occurs to some degree with all resorbable materials. Inflammation only becomes a problem if it becomes prolonged (chronic) and increases in severity leading to immunologically mediated events that lead to destruction of the implants or cell/tissue death. Reviews on the inflammatory response to biomaterials are available (Anderson et al., 2008 and Slee et al., 2014) and will not be discussed here.

### Biomaterials and Biodevices-Related Infections

As foreign materials that have been introduced into the body, biomaterials would also be a potential source of infection. Biomaterials made from metals, ceramics, and polymers are now in routine clinical use and have been linked to infection (Buhmann et al., 2016 and Busscher et al., 2012). For example, approximately 60,000 deaths per year have been reported in the USA due to device-related infections from urinary catheters and central venous catheters, and those made from polyurethane have been shown to constitute an entry pathway into body for bacteria (O’Grady et al., 2002). Bacteria will compete with cells to adhere to surface of biomaterials, as many of them have similar mechanism of attachment as cells, except they are better adapted for survival on non-viable surfaces. Common bacterial infections on polymeric biomaterials come from *Staphylococcus epidermidis* (*S. epidermidis)* from skin and *Staphylococcus aureus* (*S. aureus*), which is often found on metallic biomaterials. Some of these bacteria may be resistant to antibiotics (different surface expression). These have been found on artificial hearts, synthetic vessels, joint replacement implants, fixation devices, IV catheters, urologic devices, and contact lenses (Holzapfel et al., 2013). Ceramics and metals are relatively resistant to infection, but if there are imperfections on the surface or microfractures, pathogens, such as bacteria, can establish a colony (Holzapfel et al., 2013).

A group of aliphatic polymers, such as polyethylene, polytetrafluoroethylene, polypropylene, and also polyvinylidene fluoride, have selective affinity toward endotoxins (Davies, 1999). Thus, they have the potential to facilitate the microenvironment for tissue regeneration by adsorbing the endotoxins. However, due to absence of hydrophilic ionizable groups, they cannot be used directly before further biocompatible functionalization. Charged polymers with effective functional groups can selectively bind and remove endotoxins from the systems. One such example is positively charged acrylic cellulose with DEAE or QAE functional groups that can significantly absorb endotoxins (Hou and Zaniewski, 1990). This could be an important aspect while designing advanced biomaterials for clinical translations.

### Extracellular Matrix and Pathogenic Transmission

More recently, biomaterials from natural sources have gained significant interest as scaffolds for promoting regeneration. In particular, the decellularization of organs and tissues to obtain scaffolds composed extracellular matrix (ECM) components have gained considerable popularity (Faulk et al., 2014). Such scaffolds have been shown to be conducive to regeneration. However, these are either derived from human cadaveric sources or from xenogeneic sources. Both sources carry a risk for pathogenic transmission. Xenogeneic scaffolds have an additional risk of inducing allergic or inflammation reactions. Bone allografts are known to transmit several deadly viruses like hepatitis, tuberculosis (TB), and human immunodeficiency virus (HIV-1) (Vincent, 2012). Similarly, corneas have been reported to transmit hepatitis B virus (HBV), rabies, cytomegalovirus (CMV), Creutzfeldt–Jakob disease (CJD), and herpes simplex virus (HSV), apart from different bacteria and fungi (Lee et al., 2007). Furthermore, heart valves have been shown to transmit TB and HBV (Zou et al., 2004). Skins from seropositive donors were associated with HIV-1 and CMV transmission (Eastlund, 1995). Therefore, critical care and appropriate safety measures, such as rigorous screening, are required during the process of transplanting decellularized organs.

## Biomaterials That Modulate Inflammation

Despite being necessary during the early stages of wound healing, inflammation plays a major role in the rejection of biomaterial implants. Dysregulated and excessive or chronic inflammation has a negative impact on the wound healing processes. Therefore, strategies to modulate excessive inflammation are needed. Through the use of biomaterials to control the release of anti-inflammatory therapeutics, increased control over inflammation is possible in a range of pathological conditions. However, the choice of biomaterial (natural or synthetic) and its form (solid, hydrogel, or micro/nanoparticle) is dependent on both the cause and tissue location of inflammation. These considerations also influence the nature of the anti-inflammatory therapeutic that is incorporated into the biomaterial to be delivered. There are two groups of biomaterials: those that possess intrinsic anti-inflammatory properties and those that are designed to incorporate anti-inflammatory agents. Examples of each group are given below.

### Biomaterials with Intrinsic Anti-inflammatory Properties

Some biopolymers, and in particular, polysaccharides, have inherent anti-inflammatory properties. For example, chitosan, a linear polysaccharide composed of randomly distributed β-(1–4)-linked d-glucosamine and *N*-acetyl-d-glucosamine derived from crustacean shells, has long been reported to have anti-inflammatory properties. Song et al. studied the anti-inflammatory effects of the chitosan–gelatin hybrid materials cross-linked with genipin. They concluded that the anti-inflammatory effects of genipin could be due to its effect on the NO/iNOS pathway and inhibition of the mRNA expression of COX-2 and IL-6 within activated macrophages (Song et al., 2011). Chitosan-based materials are believed to be anti-inflammatory based on their ROS scavenging properties (Je and Kim, 2006). Other biopolymers, primarily polysaccharides from plants, such as mushrooms (Elsayed et al., 2014) and seaweed (Rodrigues et al., 2012 and Park et al., 2011), are being examined for their anti-inflammatory properties. It is possible for these in the future to be tested as biomaterials.

Polyethylene glycol (PEG) and its nano-conjugated derivatives have also been shown to possess anti-inflammatory properties (Dobrovolskaia and McNeil, 2007). For example, PEG has been hybridized by incorporation of peptides, such as GRGDSPG, to form hydrogels with anti-inflammatory properties. GRGDSPG-containing peptides have been reported to protect MIN6 mouse pancreatic islet-derived cells from cytokine-induced cell death when functionalized to encapsulate these cells. These peptide-containing hydrogels in conjunction with the interleukin-1 receptor inhibitory peptide (IL-1RIP) FEWTPGWYQPY-NH2 were particularly effective in protecting the islet cells (Su et al., 2010).

### Integrative Anti-inflammatory Materials

Implantation of tectonic patches made from interpenetrating networks of collagen and 2-methacryloyloxyethyl phosphorylcholine (MPC) into three patients in a small hospital-based study under compassionate use revealed that this material was able to stably restore the integrity of the damaged corneas of patients with chronic ulceration or erosion of the epithelium due to stroma damage, thereby relieving patients from pain, discomfort, and photophobia (Buznyk et al., 2015). MPC has also been shown to have anti-inflammatory properties in other systems. For example, MPC-polymer has been shown to be useful for oral care. It protects from oral infection by preventing the adherence of periodontal pathogen and succeeding inflammatory reaction and, thus, protects gingival epithelium to maintain oral epithelial function (Yumoto et al., 2015).

Hyaluronic acid (HA) hydrogels have been widely used as scaffolds for promoting regeneration because of their reported anti-inflammatory nature (Nakamura et al., 2004; Hirabara et al., 2013). TNF-α antibody-conjugated HA hydrogels have been shown to reduce IL-1β concentration and macrophage infiltration when applied to burn wounds. This, in turn, reduced the thickness of the non-viable tissue (Friedrich et al., 2014). HA-based scaffolds with mesenchymal stem cells completely eliminated the inflammatory process when transplanted in pig models of myocardial infarction (Muscari et al., 2013).

To reduce the foreign body reaction (FBR), plasmid-encoded or virus-encapsulated IL-10 can efficiently downregulate the inflammatory response against collagen scaffolds, when transplanted subcutaneously in rats (Van Putten et al., 2009; Holladay et al., 2012). Combined glycosaminoglycan high sulfated hyaluronan with collagen scaffold reduced the secretion of pro-inflammatory cytokines and increased the anti-inflammatory cytokines, when macrophages were cultured on the implants (Kajahn et al., 2012) in *in vitro*-mimicked conditions of sterile tissue injury.

### Biomaterials Developed as Delivery Systems to Control Inflammation

Not all biomaterials in clinical use are inherently anti-inflammatory in nature. However, they have been used to deliver a wide variety of therapeutic agents that were developed to control inflammation. The need for delivery agents stems from the fact that most therapeutic agents on their own fail to achieve a high enough local concentration to exert their effects. One approach to address this problem is the use of biomaterials as delivery agents and reservoirs for the therapeutic agent(s) and, in particular, provide sustained release of effective concentrations for prolonged periods of time. For example, the incorporation of stromal cell-derived factor-1 alpha (SDF-1α) into PLGA scaffolds reduced inflammation when transplanted into the subcutaneous cavity of Balb/C mice to improve both the tissue response and regenerative potential of tissue engineering scaffolds by reducing the local inflammation. It decreased the number and responses of mast cell near the implants together with reduced expression of IL-1α, IL-6, and TNF-α and increased VEGF expression (Thevenot et al., 2010).

A PLLA scaffold releasing Ibuprofen was shown to reduce IL-6 and TNF-α expression leading to decreased inflammatory responses and improved muscle regeneration (Yuan et al., 2014). Dexamethasone incorporated hydrogels reduced TNF-α and IL-6 expression from macrophages that ultimately reduced inflammatory response in lipopolysaccharide-stimulated primary mouse macrophages *in vitro* (Ito et al., 2007). Gelatin hydrogels incorporating mixed immunosuppressive triptolide-micelles and bone morphogenic protein-2 (BMP-2) downregulated the expression of pro- and anti-inflammatory cytokines, including IL-6 and IL-10, and reduced local inflammation responses and enhanced bone regeneration in rat model (Ratanavaraporn et al., 2012).

Although a plethora of potential delivery systems have been reported, only a few anti-inflammatory drug delivery materials are currently used in the clinic. One of these is a system for sustained delivery of dexamethasone (Ozurdex, Allergan Inc., Irvine, CA, USA). The implant is introduced into the posterior segment of the patient’s eyes with various pathologic conditions, including diabetic macular edema, non-infectious intermediate uveitis, and birdshot chorioretinopathy, and has been shown to exhibit a good safety profile and promising results in edema and inflammation control (Cao et al., 2014; Dugel et al., 2015; Walsh and Reddy, 2016). Further clinical trials are ongoing to confirm these initial results (ClinicalTrials.gov identifier: NCT01801774, NCT02736175, and NCT02547623).^2^

## Biomaterials That Modulate Infection

### Biomaterials with Intrinsic Anti-infective Properties

Just like there are biomaterials with innate anti-inflammatory behavior, there are biomaterials that have intrinsic anti-infective properties, and there are those that are effective as carriers of antibacterial and antiviral agents or other bioactives developed to combat infectious agents. There is a wide range of these and only a selected few examples are provided below. Biomaterials containing sulfated groups are known to have anti-infective properties. These include antibacterial as well as antiviral properties. The best-known are the marine-derived sulfated polysaccharides derived from brown seaweeds (*Phaeophyceae* such as *Fucus, Laminaria*, and *Ascophyllum*). These macromolecules include alginates and fucoidans (Berteau and Mulloy, 2003; Marguerite, 2014).

Among the properties attributed to fucoidans is its antiviral activity (Damonte et al., 2004). Fucoidans isolated contain mainly O-sulfated α-l-fucosides but they also contain acetyl groups and other types of saccharides and proteins (Morya et al., 2012). Our group had examined the possibility of reproducing the antiviral properties of fucoidans in synthetic mimics and confirmed that the sulfation was essential for activity against viruses, such as Herpes Simplex Virus serotype 1 (HSV-1) (Tengdelius et al., 2014). However, we also found that the activity of synthetic fucoidan was similar to that of other sulfated polysaccharides, such as heparin and dextran sulfate, while non-sulfated control synthetic fucoidans or polyacrylamide did not block viral activity. We, further, showed that synthetic O-sulfated fucoidans blocked HSV-1 activity during the viral adsorption step, reacting with viral particles to prevent their entry into cells (Tengdelius et al., 2014). More recently, we examined the anti-HSV-1 efficacy of another fully synthetic sulfated biomaterial, polystyrene sulfonate [poly(sodium 4-styrenesulfonate) (PSS)]. We developed theranostic contact lenses, i.e., contact lenses that could detect and modulate HSV-1 infection. Here, PSS was used as coatings to effectively provide antiviral activity (Mak et al., 2015).

### Nanomaterials with Antimicrobial Properties

A wide range of nanomaterials, from carbon nanotubes (CNTs) and fullerenes to dendrimers and metal nanoparticles has been shown to have intrinsic anti-infective properties against bacteria, viruses, and other pathogens (reviewed in Rai et al., 2015). Table 1 provides a list of various nanoparticle systems and their reported antimicrobial (antibacterial) activities.

**Table 1:** **Type of various nanoparticles and their applications in antimicrobial and antiviral treatment**.

Type of nanoparticles	Antimicrobial activity	Reference
***Metallic nanoparticles***		
ZnO, CuO, Fe_2_O_3_	*E. coli, P. aeruginosa, S. aureus, B. subtilis*	Azam et al. (2012)
Silver, titanium dioxide, silica dioxide nanoparticles	*S. mutans*	Besinis et al. (2014)
Sliver	*E. coli, P. aeruginosa, S. aureus*	Guzman et al. (2012)
Silver (biological)	*E. coli, S. aureus, E. faecalis*	Barbinta-Patrascu et al. (2014)
Silver (biological and chemical)	*E. coli, S. aureus*	Bawskar et al. (2015)
Collagen-AgNP hydrogels	*S. aureus, S. epidermidis, E. coli, P. aeruginosa*	Alarcon et al. (2015)
***Carbon nanotubes (CNTs)***		
Single/multi-walled CNTs	*L. acidophilus, B. adolescentis, E. coli, E. faecalis, S. aureus*	Chen et al. (2013)
AgNPs decorated CNTs	*E. coli, S. aureus*	Dinh et al. (2015)
Chitosan-CNT hydrogels	*S. aureus, E. coli, C. tropicalis*	Venkatesan et al. (2014)
Gelatin-CNTs	*K. pneumoniae, E. coli*	Spizzirri et al. (2015)
*Functionalized fullerenes*	*E. coli, C. albicans, S. aureus*	Mizuno et al. (2011); Tegos et al. (2005)
*Dendrimers*	*S. aureus, P. aeruginosa, E. coli*	Lind et al. (2015)

**Type of nanoparticles**	**Antiviral activity**	**Reference**

***Silver nanoparticles (AgNPs)***		
AgNPs	HIV-1 inhibition	Elechiguerra et al. (2005); Gaikwad et al. (2013); Hu et al. (2014); Lara et al. (2010)
PVP-stabilized AgNPs	HSV and HPIV	
Mercaptoethanesulfonate-capped AgNPs	HSV-1	Baram-Pinto et al. (2010)
AgNPs/chitosan composites	H1N1 influenza	Xiang et al. (2011); Mori et al. (2013)
Polysaccharide-coated AgNPs	Tacaribe virus	Speshock et al. (2010)
***Gold nanopartices (AuNPs)***		
Glucose-coated AuNPs	HIV treatment	Chiodo et al. (2014)
AuNPs conjugated with peptide triazoles	HIV treatment	Bastian et al. (2015)
Sulfated ligands-coated AuNPs	HIV treatment	Di Gianvincenzo et al. (2010)
Fluorescein-labeled oligomannoside AuNPs	HIV treatment	Martínez-Avila et al. (2009); Arnáiz et al. (2012)
Mercaptobenzoic acid-coated AuNPs	HIV treatment	Bowman et al. (2008)
Mercaptoethane sulfonate-capped AuNPs	HSV-1	Baram-Pinto et al. (2010)
*Solid lipid nanoparticles (SLN)*	Hepatitis B virus (HBV)	Zhang et al. (2008)
*Quantum rods (QRs)*	HIV-1	Mahajan et al. (2010)

The best-known metallic nanoparticles with antibacterial activity are the silver nanoparticles (AgNPs) and silver in general. These are known for their antibacterial and antiviral properties. Use of silver in medical implants has a long history of medical use. Colloidal silver has been approved for wound treatment since the 1920s and registered as a bactericidal substance since 1954 (Nowack et al., 2011; Reidy et al., 2013). New solutions combine regeneration and antimicrobial effects to allow faster and safer recovery from injury. Silver containing wound dressings include Acticoat^®^, a commercial wound dressing utilizing nanocrystaline silver (Khundkar et al., 2010), Actisorb^®^, which contains a silver-nylon cloth, and Calgitrol Ag^®^ that utilizes silver-alginate (Simon et al., 2016). Silver ions have been shown to have toxicity on cells and also *in vivo*. However, it has been shown that silver nanoparticles produced as naked particles and coated with collagen or LL-37 peptide have reduced cytotoxicity on human skin epidermal cells compared to ionic silver (Alarcon et al., 2015). However, they were effective against bacteria tested, such as *S. aureus* (strain ATTC 25923), *S. epidermidis* (strain Se19), *Escherichia coli* (strain CFT073), and *Pseudomonas aeruginosa* (strain PA01). Methicillin-resistant *S. epidermidis* (MRSE) and methicillin-resistant *S. aureus* (MRSA) have been reported to be successfully inhibited by “NanoSilver” embedded bone cement in an *in vitro* model with human osteoblasts, whereas similar cement with gentamicin could not able to prevent the infection with such resistant strains (Alt et al., 2004). Within our group, we have developed simple composite collagen-based hydrogels that have included silver nanoparticles with antibacterial properties as corneal implants (Alarcon et al., 2016).

### Antiviral Nanosystem

Nanoparticles and their different composites have also had antiviral activity (Table 1). They have now been shown to interact with the HIV-1 virus in a size-dependent manner possibly through the gp120 subunit of the viral envelope glycoprotein (Di Gianvincenzo et al., 2010; Lara et al., 2010).

Like antibiotics, however, resistance to silver has been found in bacteria. The reports, to date, point to the resistance being plasmid-based, and not all bacteria examined have been shown to harbor these plasmids. Overall, the incidence of silver resistance remains low compared to antibiotic resistance (Griffith et al., 2015).

### Composite Biomaterials as Delivery Systems

Nanoparticles have also been used as carriers for bioactives and antiviral drugs. For example, silver and gold coated with sulfated ligands developed have been shown to exert their anti-HIV activity by inhibiting the binding of HIV gp120 on the host cell receptors at early stage of viral replication (Di Gianvincenzo et al., 2010; Lara et al., 2010). Several studies have evaluated the potential antiviral efficacy of antiviral drug delivery systems against HIV-1 infection (Bowman et al., 2008; Mahajan et al., 2010; Chiodo et al., 2014; Bastian et al., 2015) or HBV, showing promising results in terms of enhanced antiviral potency or efficiency in delivery of the drugs/peptides used. Zhang et al. showed that adefovir dipiroxil (ADV), a nucleotide analog with potent antiviral activity against chronic HBV, loaded in solid lipid NPs significantly lowered HBV DNA levels compared with free ADV (Zhang et al., 2008).

We have previously reported collagen implants containing silica nanoparticles releasing LL37 peptide that has anti-HSV-1 antiviral properties (Lee et al., 2014). Other more sophisticated composite biomaterials have been developed as local antibiotic carriers system that offer regulated release of antibiotics in specific tissues and implant. An example of such biomaterials is TiO_2_-NiFe_2_O_4_ nanoparticle system that comprise particles with photocatalytic shells and magnetic cores, to form removable antimicrobial photocatalyst system that can be extracted from the sprayed surface (infected region) after exposure (Rana et al., 2005). Biocompatible, injectable polymeric carriers, e.g., Pluronic^®^ F127 that can respond *in situ* to physiological stimuli have also been developed for controlled drug release (Simões et al., 2012). To combat with the increasing concern of antibiotic resistance, composite nanosystems that combine conventional antibiotics with nanoparticles have been reported to successfully inhibit drug resistant microbes compared with antibiotics alone (Campoccia et al., 2010). Several approaches are going on with antibiotic-loaded biomaterials for local infection prophylaxis, and one such example that is available for use in the clinic is poly(d,l-lactide) (PDLLA) coating. The idea is to turn an implant into a drug delivery device.^3^

**Figure 1:**
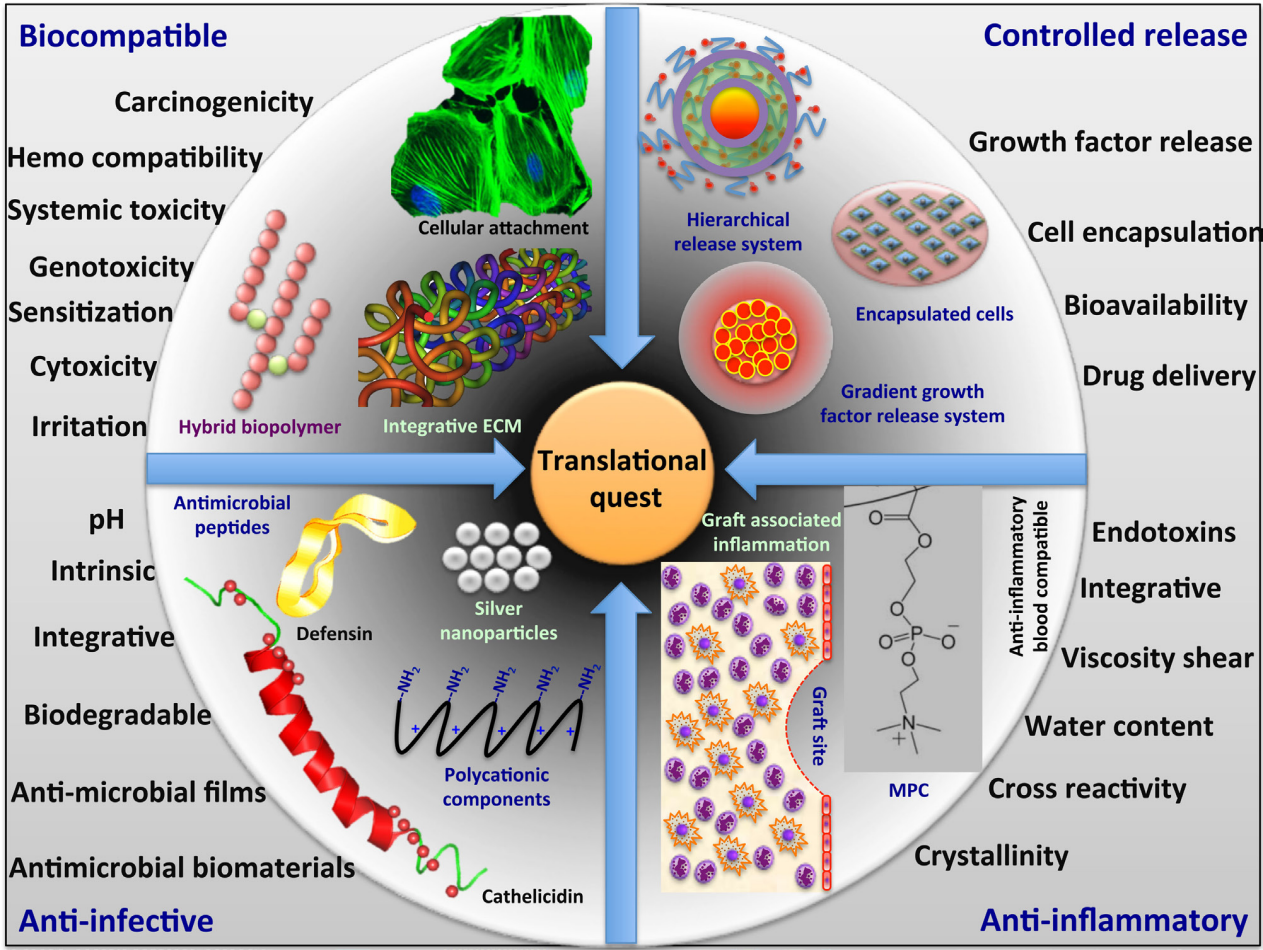
**Translational quest for next generation biomaterials**.

We have summarized the different functional aspects and the major concern in developing the biomaterials for their successful clinical translation (Figure 1).

## Conclusion

In conclusion, biomaterials are now being developed to address issues of infection and inflammation in compromised patients. These include those materials with inherent anti-inflammatory or anti-infective properties as well as materials bioengineered to deliver those properties. Several examples of each have now reached the pre-clinical and clinical evaluation stages and show promising results. However, this area is still in its infancy, and the search for biomaterials and implants that can promote regeneration while addressing localized infection and inflammation continues.

## Author Contributions

HP and MG conceptualize the area for this review. MI, JE, GP, and OB have contributed in their respective sections.

## Conflict of Interest Statement

The authors declare that the research was conducted in the absence of any commercial or financial relationships that could be construed as a potential conflict of interest.

## Footnotes

^1^http://www.organdonor.gov/about/data.html

^2^https://clinicaltrials.gov/

^3^www.synthes.com
